# Management of recurrent pleural mesothelioma: Successful rechallenge with nintedanib in combination with chemotherapy

**DOI:** 10.1002/ccr3.1775

**Published:** 2018-09-04

**Authors:** Federica Grosso, Annalisa Roveta, Giulia Gallizzi, Marco Belletti

**Affiliations:** ^1^ Mesothelioma Unit SS Antonio e Biagio General Hospital Alessandria Italy; ^2^ Clinical Trial Center SS Antonio e Biagio General Hospital Alessandria Italy; ^3^ Department of Oncology Santo Spirito Hospital Casale Monferrato Italy; ^4^ Department of Radiology SS Antonio e Biagio General Hospital Alessandria Italy

**Keywords:** anti‐angiogenic, disease progression, malignant pleural mesothelioma, nintedanib

## Abstract

Malignant pleural mesothelioma (MPM) is a rare neoplasm, generally caused by asbestos exposure. This case details how a patient treated with nintedanib during the LUME‐Meso study was rechallenged with nintedanib. The findings highlight the benefit of nintedanib rechallenge and the potential use of continuous anti‐angiogenic therapy in MPM treatment.

## 1 INTRODUCTION

Malignant pleural mesothelioma (MPM) is a rare, aggressive neoplasm that is usually diagnosed at an advanced stage with an unfavorable prognosis.^1^, ^2^ Eighty percent of cases are caused by industrial exposure to asbestos.

For patients with unresectable disease, systemic first‐line treatment with pemetrexed and cisplatin is the only regimen that has been approved by the European Medical Association^3^ and the Food and Drug Administration, and recommended by the National Comprehensive Cancer Network (NCCN).^4^ Other acceptable first‐line combination chemotherapy options recommended by the NCCN include bevacizumab, cisplatin, and pemetrexed; pemetrexed/carboplatin; and gemcitabine/cisplatin. Similarly, the European Society for Medical Oncology guidelines suggest combination doublet chemotherapy of cisplatin with either pemetrexed or raltitrexed. Carboplatin is an acceptable alternative to cisplatin and may be better tolerated in the elderly population.^5^

There are currently no approved agents for second‐ or third‐line treatment.^5^ Vinorelbine monotherapy, pemetrexed plus cisplatin, and nivolumab plus ipilimumab have shown activity in Phase II and Phase III studies in this setting.^6^, ^7^, ^8^ Studies with small molecule, multitargeted tyrosine kinase inhibitors, however, have been unsuccessful in demonstrating sufficient clinical response.^9^, ^10^, ^11^, ^12^ In this respect, European guidelines recommend that, in the absence of standard second‐ or further‐line therapy, patients are enrolled into clinical trials.^5^ There have since been few advances in treatment.^13^ The exception is the MAPS Phase III trial, investigating the addition of bevacizumab to pemetrexed/cisplatin.^14^, ^15^ Additionally, the efficacy and safety of nintedanib plus chemotherapy as first‐line treatment are being evaluated in the LUME‐Meso Phase II/III randomized, double‐blind study (NCT01907100).^16^ In the Phase II portion of the trial, the addition of nintedanib to pemetrexed/cisplatin yielded improvement in progression‐free survival (the primary endpoint) and a trend toward improved overall survival.^16^ The Phase III portion of this study is still ongoing.

## 2 CASE

In January 2014, a 53‐year‐old man experienced dyspnea induced by medium intensive efforts. His comorbidities included essential hypertension and gastroesophageal reflux disease (GERD). In February 2014, the patient reported left chest pain, which was initially attributed to GERD. Past medical history revealed no history of smoking. As a factory worker, however, he had been exposed to environmental asbestos.

**Figure 1:**
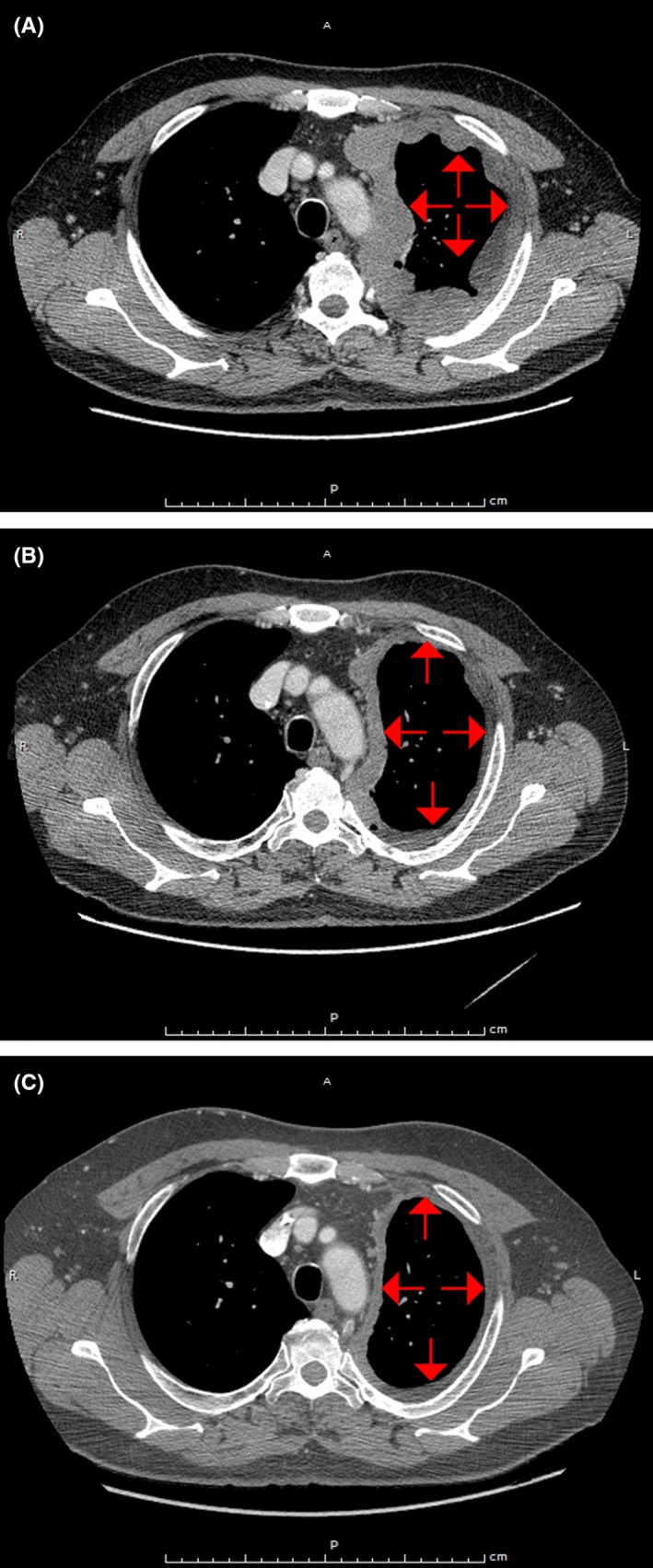
Disease reduction following nintedanib treatment. A, CT scan prior to nintedanib plus pemetrexed treatment. B, CT scan following 38 d of nintedanib treatment (plus pemetrexed plus cisplatin). C, CT scan following 129 d of nintedanib treatment (as maintenance therapy). Arrows show location of neoplasm. CT, computed tomography; d, days

In March 2014, results of the chest radiograph showed massive left pleural effusion. One week later, a computed tomography scan of the chest revealed a large mediastinal, parietal, and diaphragmatic left pleural thickness, along with paratracheal and contralateral enlarged right hilar lymph nodes (Figure 1A). In April 2014, the patient underwent left video‐assisted thoracoscopic surgery with talc pleurodesis for pleural effusions.

Histologic analysis of the three pleural biopsies revealed morphology that was consistent with epithelioid subtype MPM. At baseline, the total tumor measurement of target lesions (according to modified Response Evaluation Criteria in Solid Tumors [RECIST])^17^ was 116 mm, and the forced vital capacity (FVC) was 2.57 L.

**Figure 2:**
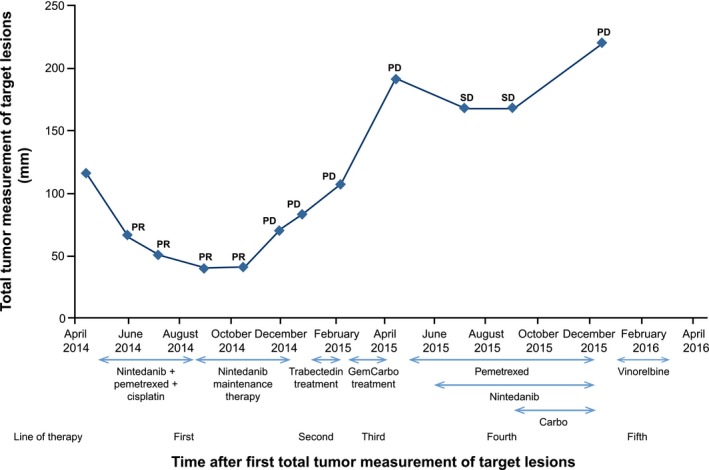
Summary of treatment course. PD equates to ≥20% taking as reference the nadir; PR equates to >30% decrease in total tumor measurement from baseline; SD equates to neither PD nor PR criteria. Carbo, carboplatin; GemCarbo, gemcitabine plus carboplatin; PD, progressive disease; PR, partial response; SD, stable disease

**Figure 3:**
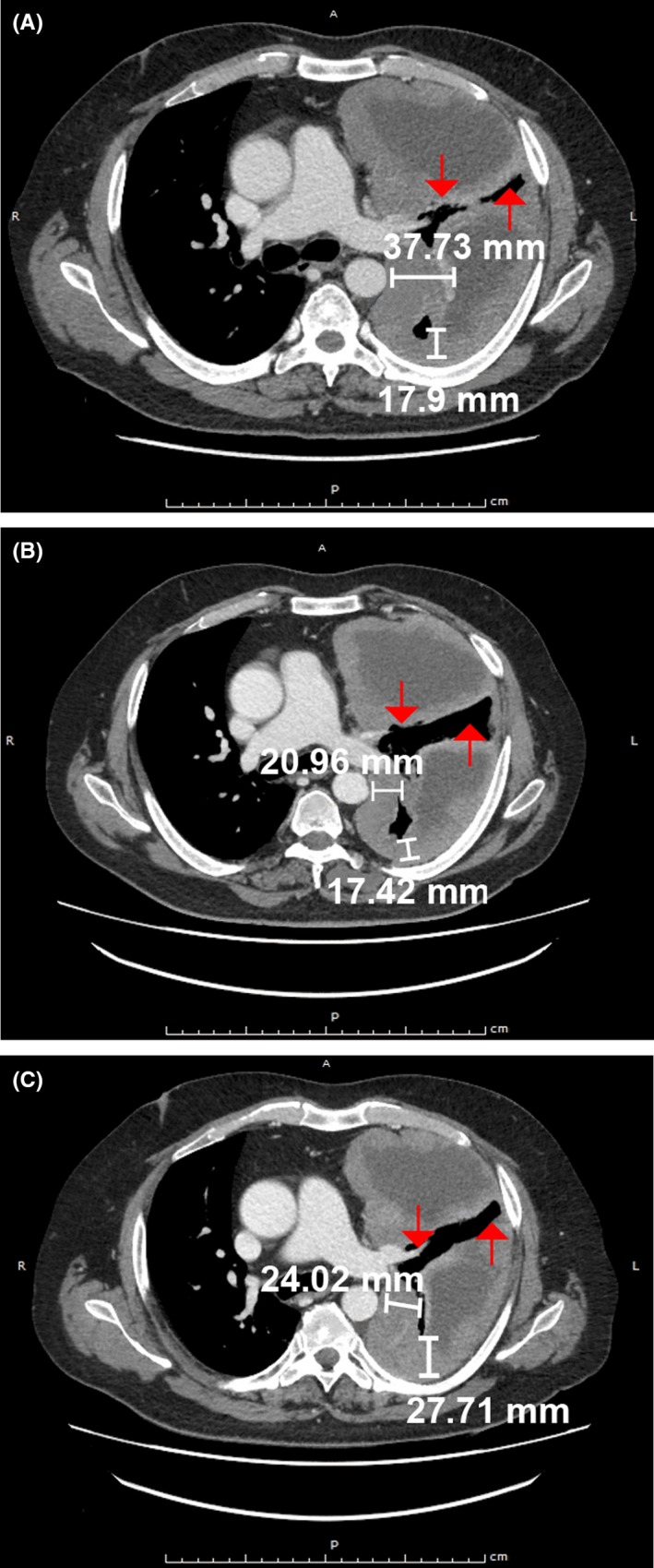
Disease reductions following nintedanib plus pemetrexed rechallenge. A, CT scan prior to nintedanib plus pemetrexed rechallenge. B, CT scan following 59 d of nintedanib plus pemetrexed rechallenge. C, CT scan following 116 d of nintedanib plus pemetrexed rechallenge. Arrows show location of neoplasm. CT, computed tomography; d, days

The overall course of treatment is described in Figures 1, 2, 3. Following the initial diagnosis, the patient was enrolled in the LUME‐Meso Phase II randomized, double‐blind study. Although it was not known at the time, the patient was randomized to receive nintedanib in addition to pemetrexed plus cisplatin in April 2014. Pemetrexed plus cisplatin was administered for six 21‐day cycles at standard doses: 500 mg/m^2^ of pemetrexed administered intravenously (IV) over 10 minutes on Day 1, and 75 mg/m^2^ of cisplatin administered IV over 2 hours on Day 1. Nintedanib was given orally at 200 mg twice daily (bid) on Days 2–21 of each 21‐day cycle. The patient also received maintenance treatment from August 2014 to December 2014. In June 2014, during the third treatment cycle, Grade 3 venous thromboembolism was reported. The event was considered unrelated to study treatment and did not require any change to treatment.

Follow‐up imaging was carried out every 6 weeks from randomization. From June 2014 to October 2014, the patient showed a very good partial response (Figure 2), as evidenced by a decrease in the total tumor measurement of target lesions from baseline (116 mm) to 64 mm, 50 mm, 40 mm, and 41 mm at Week 6, Week 12, Week 18, and Week 24, respectively. There was an increase in FVC from baseline, as measured in May (24.1%), June (22.6%), July (16.0% and 21.0%), August (19.1%), September (15.6%), October (21.4% and 25.7%), and November 2014 (25.3%). In December 2014, there was tumor progression, as indicated by an increase of 77.5% in the total tumor measurement of target lesions from the nadir at Week 24. In the same month, the change in FVC from baseline was 14.4%. Treatment was subsequently stopped due to disease progression that was seen in the final tumor assessment. Following discontinuation, the patient reported an increase in symptoms such as thoracic pain and dyspnea during tasks requiring mild effort.

In January 2015, the patient was enrolled in the Phase II ATREUS study, at which time the total tumor measurement of target lesions was 92 mm. The patient received 1.1 mg/m^2^ IV trabectedin infusion in 5% glucose via central venous catheter over 3 hours every 21 days. The trabectedin infusion was preceded by 20 mg of IV dexamethasone. The best response to trabectedin was progressive disease (total tumor measurement of target lesions: 107 mm). In February 2015, third‐line treatment with gemcitabine plus carboplatin was administered for three cycles but, again, showed progressive disease (Figure 3A).

In May 2015, the patient was rechallenged with six cycles of fourth‐line pemetrexed, with the addition of nintedanib 200 mg bid from the second cycle onwards. Treatment was received through individual compassionate use. This regimen stabilized disease progression again for >5 months (Figure 2), with the sustained response indicated by a decrease of 12.6% in the baseline tumor measurement between July 2015 and September 2015 (Figures 2 and 3B). This disease stabilization was associated with a clear clinical benefit in terms of symptom relief and improved dyspnea. During the rechallenge, the patient reported a Grade 2 perimalleolar edema, for which furosemide was administered at 10 mg per day. Although the patient showed stable disease according to RECIST, a slight increase in target lesions was observed in September 2015 (Figure 3C), and therefore, carboplatin was added to the treatment regimen; however, disease progression continued. In December 2015, the patient's total tumor measurement of target lesions had increased to 219 mm, with new nontarget lesions observed.

At the end of December 2015, the patient was administered three cycles of fifth‐line vinorelbine; however, the disease continued to progress and the patient died at the end of March 2016, 23 months after the diagnosis of MPM.

## 3 DISCUSSION

MPM is a challenging tumor to diagnose and to treat. Moreover, its clinical course is aggressive, with a short survival time.^1^ This case report shows a substantial clinical benefit experienced by a relatively young patient during first‐line treatment with nintedanib in combination with chemotherapy. Disease progression continued rapidly after discontinuation of first‐line combination treatment, and the patient showed no response to second‐ or third‐line therapies. This individual case is notable because, when the patient was subsequently rechallenged with nintedanib in combination with chemotherapy, the disease stabilized for >5 months. In our opinion, the clinical benefits (coupled with improved quality of life) reported by the patient with nintedanib in combination with chemotherapy are not observed with standard chemotherapy.

A key factor related to the successful rechallenge in this patient appears to be the influence of the patient's successful first response to nintedanib combined with pemetrexed and cisplatin. While there are no clinical data indicating the benefits of continued therapy with nintedanib, previous research has shown that patients who have benefited from pemetrexed‐based therapy in the first‐line setting can benefit from retreatment in a second or later line.^18^, ^19^

The benefit of nintedanib rechallenge may support the concept of continuous anti‐angiogenic therapy as a treatment strategy in MPM. Critically, further research is needed to evaluate the clinical impact of such a strategy.

## 4 CONCLUSION

This case study highlights the need for effective treatments for MPM after first‐line therapy. Nintedanib in combination with chemotherapy was effective as first‐line therapy and provided sustained disease stabilization on rechallenge 6 months after initial nintedanib therapy had ended. Furthermore, nintedanib in combination with chemotherapy showed acceptable tolerability, as observed by the low incidence of reported adverse events.

## CONFLICT OF INTEREST

Federica Grosso has received travel, accommodation, and expense support from Boehringer Ingelheim. Annalisa Roveta, Giulia Gallizzi, and Marco Belletti have no conflict of interests.

## AUTHORSHIP

FG, AR, GG, and MB: had full access to all of the data in the study, took responsibility for the integrity of the data and the accuracy of the data analysis, and were involved in acquisition, analysis, and interpretation of data. FG, AR, GG, and MB: drafted the manuscript and were major contributors to the manuscript.
